# Supplementary material for: Infections Caused by Moellerella wisconsensis: A Case Report and a Systematic Review of the Literature
Source: Microorganisms. 2022 Apr 24;10(5):892. doi: 10.3390/microorganisms10050892 (PMC9147471; doi:10.3390/microorganisms10050892)
Supplement: Supplementary file 1 [file microorganisms-10-00892-s001.zip › microorganisms-1657008-supplementary.pdf]

*Supplementary material*

# Infections Caused by *Moellerella wisconsensis*: A Case Report and a Systematic Review of the Literature

**Table S1.** Antibiogram.

| Culture: Blood Culture | | |
|---|---|---|
| Isolated Microbe: *Moellerella wisconsensis* | | |
| **Antibiotic** | **Antibiotic Sensitivity** | **M.I.C.** |
| Amikacin | R | >32 |
| Amoxicillin/Clavulanate | S | ≤4/2 |
| Ampicillin | R | >4 |
| Cefepime | R | >16 |
| Cefoxitin | R | >16 |
| Ceftazidime | R | >16 |
| Ceftriaxone | R | >32 |
| Cefuroxime | R | >16 |
| Ciprofloxacin | R | >2 |
| Colistin | R | >2 |
| Ertapenem | R | >4 |
| Gentamicin | R | >8 |
| Imipenem | S | ≤2 |
| Levofloxacin | R | >4 |
| Meropenem | S | ≤4 |
| Piperacillin/Tazobactam | S | 8/4 |
| Tigecycline | S | ≤1 |
| Trimethoprim/Sulfamethoxazole | R | >4/76 |

R: Resistant, S: Sensitive, I: Intermediate, M.I.C.: minimum inhibitory concentration
Same antibiograms for urine culture and pigtail end culture

**Table S2.** Reasons for exclusions of studies from the systematic review.

| Study | Reasons of Exclusion |
|---|---|
| Stock et al. [1] | Report of *Moellerella wisconsensis* case isolated from human peritoneum, but the citation could not be retrieved |
| Sandfort et al. [2] | *Moellerella wisconsensis* isolated from the oral cavity of a wildraccoon |
| Zambarbieri et al. [3] | *Moellerella wisconsensis* isolated from the urinary tract of a dog |
| Casalinuovo et al. [4] | *Moellerella wisconsensis* isolated from the lung of a goat |
| Cabadajová et al. [5] | Study could not be retrieved |

1. Stock I, Falsen E, Wiedemann B. Moellerella wisconsensis: identification, natural antibiotic susceptibility and its dependency on the medium applied. Diagn Microbiol Infect Dis. 2003;45:1-11. https://doi.org/10.1016/s0732-8893(02)00483-2
2. Sandfort RF, Murray W, Janda JM. Moellerella wisconsensis isolated from the oral cavity of a wild raccoon (Procyon lotor). Vector Borne Zoonotic Dis. 2002;2:197-9. https://doi.org/10.1089/15303660260613765
3. Zambarbieri J, Grilli G, Vitiello T, Scarpa P. Urinary tract infection by atypical uropathogens in dogs. Vet Ital. 2021;57:89-92. https://doi.org/10.12834/VetIt.2110.12149.1
4. Casalinuovo F, Musarella R. Isolation of Moellerella wisconsensis from the lung of a goat. Vet Microbiol. 2009;138:401-2. https://doi.org/10.1016/j.vetmic.2009.03.028
5. Cabadajova D, Kudrna L. [Moellerella wisconsensis--the first isolation and identification of a new genus and species of the family Enterobacteriaceae in Czechoslovakia]. Cesk Epidemiol Mikrobiol Imunol. 1988;37:45-8.
